# Effect of hypospadias on sexual function and reproduction

**DOI:** 10.4103/0970-1591.40623

**Published:** 2008

**Authors:** J. Chandra Singh, Venkata Rama Jayanthi, Ganesh Gopalakrishnan

**Affiliations:** Department of Urology, Christian Medical College, Vellore, Tamil Nadu, India; 1 Division of Pediatric Urology, Nationwide Children's Hospital, Columbus, OH, USA

**Keywords:** Hypospadias, reproduction, sexual function

## Abstract

Hypospadias is a highly prevalent congenital anomaly. The impact of the defect and operative interventions on sexual and reproductive function has been addressed by few publications. It is essential to know the possible outcomes of intervention for appropriate counseling, operative planning, and follow-up. English articles indexed in Pubmed dealing with the long-term sexual and reproductive outcome following hypospadias repair from 1965 to 2007 were reviewed. To our knowledge, there was no prospective trial comparing the impact of various techniques on sexual outcome. There is considerable discordance in literature regarding the effects on sexual function. A few publications report patient and partner dissatisfaction with the appearance of genitalia. Sexual dissatisfaction is often attributed to penile size. Ejaculatory disturbances range between 6 and 37% of operated individuals. There is no convincing evidence for impaired fertility. The long-term follow-up is essential to identify problems and to address them appropriately. Literature documenting the outcome of specific operative procedures and analysis based on severity of hypospadias will be informative. The long-term follow-up of the newer techniques which are more commonly used are awaited.

## INTRODUCTION

In hypospadias, the inherent difficulties to reconstruct the urethra, straighten the penis, and to restore the appearance of the penis are evident from the number of techniques and modifications described in the literature. However, the impact of the deformity extends beyond the realms of a structural defect, by virtue of the diverse functions of the penis. To counsel parents and patients appropriately, it is essential to know the effect on sexual function and reproduction. Literature on the long-term outcome, impact on sexual function and reproduction continues to be sparse. We reviewed the published literature on the sexual and reproductory outcome of hypospadias.

## GENITAL PERCEPTION

Publications on the psychological, social, and sexual development of patients operated on for hypospadias are still rare and the results are somewhat discordant. The possible explanations for these discrepancies are mainly methodological, with too small series, low rates of response to questionnaires, study populations of different ages and above all the absence of a control group, which prevents any comparison of the results with those of a reference population.[1] Another possible reason for difficulty in long-term follow-up is that the patient, after growing up to be an adult, often does not follow up with the initial surgeon. Moriya *et al.*[2] observed that the rate of dissatisfaction with penile appearance was slightly higher in the hypospadias group than in age matched controls which was not statistically significant (40.9% vs. 34.2%; *P* = 0.809). The single reason for dissatisfaction in hypospadias group was smaller penile size. Mureau *et al.*[3] interviewed 116 hypospadias patients and 88 controls who underwent hernia repair, between 9 and 18 years of age. They noted that approximately 25% of the patients reported dissatisfaction with penile appearance compared to only about 5% of the controls. Scars, penile size, and glanular shape were the most spontaneously reported reasons for dissatisfaction. Weber *et al.*[4] used a genitalia perception score (GPS) that ranged from a minimum of 1 to a maximum of 10 to evaluate self-perception of genitalia. Sixty-four patients between the age of 6 and 17 were asked to rate the appearance of their penis with regard to the following criteria: Meatus, glans, penile skin, penile straightness, and general appearance. Their GPS was almost as high as that of a control group but similar rating done by urologists on the same population was significantly less favorable. Liu *et al.*[5] observed that a higher percentage of those with proximal hypospadias were dissatisfied with the penile appearance than those who were operated for distal hypospadias (40% vs. 19.2%).

## PSYCHOSOCIAL AND SEXUAL DEVELOPMENT

Berg *et al.*[6] in the early 1980s noted that adult men operated on for hypospadias more frequently recalled depression and anxiety in addition to poorer adjustment with peers during childhood. The information pertaining to the childhood development of this group was based upon adult recall of childhood events. Hence these data might be distorted by adult experiences. Mureau *et al.*[3] compared the psychological adjustment following hypospadias surgery with a control group who underwent hernia repair. They noted that psychosocial adjustment did not significantly differ from the control group. Lesma *et al.*[7] assessed the psychosexual outcome in 30 patients using Center for Epidemiologic Studies Depression Scale (CES-D) and Self-rating Anxiety Scale (SAS) and an original structured questionnaire. Absence of depression and anxiety traits was noted in both populations. Sandberg *et al.*[8] compared 175 boys, 6- to 10-year old who underwent hypospadias repair with a community sample. Though boys with hypospadias were slightly lower in social involvement, they did not perform more poorly in schools.

### Sexual sensation

Sexual sensation has not been well documented in most articles. Bubanj *et al.*[9] noted that self-reported strength of libido was slightly better for controls compared to patients with hypospadias but without a statistically significant difference. Moriya *et al.*[2] noted that only about 10% of both patients and controls reported that their libido was low.

### Sexual life

The social and sexual life of adults operated for hypospadias during childhood has been studied by a few authors. Aho *et al.*[10] compared those who underwent hypospadias repair and circumcision. There was no significant difference in sexual and social life. Almost the same proportions reported that they were not inhibited in seeking sexual contacts. All participants reported exclusive heterosexual orientation and they were mostly satisfied with their body image. Conversely, Liu *et al.*[5] observed that 49 out of 102 patients complained that their penis had been ridiculed by partners. There was no control group in this study. They also observed that those with proximal hypospadias (60% vs. 36.5%, *P* < 0.05) and those with complications had been more often ridiculed than those in the distal group and those without complications (78.9% vs. 29.7%, *P* < 0.05). In a comparative study, Mureau *et al.*[11] compared 73 who underwent hypospadias repair in childhood with 50 controls. Though hypospadias patients reported a more negative genital appraisal than the controls, they did not have a different sexual adjustment. Higher patient age at final operation had a negative impact on sociosexual development.

## ERECTION AND SEXUAL SATISFACTION

The erectile problems in hypospadias may be attributed to surgically correctable and noncorrectable causes. More commonly encountered correctable causes include persistent chordee, torsion, inadequate cosmetic outcome, etc. Commonest surgically uncorrectable cause is the size of the penis. Achieving a straight penis is one of the objectives of hypospadias correction. With a constant move toward achieving a normal-looking penis, the results of contemporary repairs are likely to be different. Sommerlad[12] reviewed 60 adults who underwent hypospadias repair, half of which were Ombredanne repair and the unsightly redundant skin was a frequent source of complaint. Kenawi evaluated the sexual function in 82 subjects who underwent surgery for hypospadias and he noted that incomplete or incorrect surgery resulted in sexual dissatisfaction.[13] Bubanj *et al.* observed that though the frequency of intercourse during 4 weeks was significantly lesser for those who were operated for hypospadias, there were no significant difference between patients with hypospadias and controls regarding inhibition in seeking sexual contacts or patterns of sexual relationships. Those with distal hypospadias were more satisfied with their sexual life.[9] Of the 76 sexually active adults, Liu *et al.* noted that the commonest sexual complaints included short penis, increased curvature, painful erection, and no erection. The erectile problems were more in those who had proximal hypospadias.[5] They also felt that the main reason for dissatisfaction was penile size. Mureau *et al.*[3] studied noted that the lesser size of the penis was noted to be a major cause for dissatisfaction. Similar observation regarding penile size was made by Moriya *et al.*[2] Zaontz in his editorial comment of this article has underscored the importance of penile size. Sexual function and satisfaction in 10 adult patients who underwent oral buccal mucosa graft urethroplasty was studied using International Index of Erectile Functioning (IIEF-15) by Nelson *et al.* They noted that the long-term sexual function and satisfaction were excellent, in spite of them having undergone multiple procedures.[14] The long-term efficacy of dorsal plication was evaluated by Chertin *et al.*[15] Six of the 28 in whom the erection test was repeated later required further plication. Yucel *et al.* noted that 10 out of 25 who underwent re-operation for hypospadias had recurrence of chordee.[16] Bubanj *et al.* followed up patients following hypospadias repair after puberty. They found that higher number of study patients had ventral curvature during erection (40% vs. 18%) compared to controls.[9] This underscores the importance of long-term follow-up to ensure that there are no significant deformities when they become sexually active. Whether the generous utilization of procedures on the dorsal aspect of the corpora in order to preserve the urethral plate has any effects on penile length, deformity, and sexual function will be known when the long-term results of these procedures are available. A few techniques, like the one described by van der Meulen have a tendency to produce penile torsion.[17] With a decreasing trend toward using long tabularized prepucial island flaps for proximal hypospadias, penile torsion, and associated difficulty in intercourse is likely to be less. Studying whether any particular technique has an increased propensity to difficulties in erection will be informative. Tubularized incised plate (TIP), meatal advancement and glanuloplasty (MAGPI), and glanular reconstruction and prepucioplasty (GRAP) have a lesser incidence of penile torsion and the mechanical difficulties are likely to be less.

### Ejaculation after hypospadias repair

Inability to achieve satisfactory ejaculation is documented in almost all publications. Reported incidence ranges from 6 to 37%. Problems reported include weak or dribbling ejaculation, having to milk out ejaculate after orgasm, quantity of semen passing after intercourse, anejaculation with or without orgasm, etc.[1] Liu *et al.*[5] observed that the rates of ejaculation problems in the distal and proximal groups were 19.5% (8/41) and 48.6% (17/30), respectively; in the one- and two-stage groups were 16.7% (7/42) and 52.9% (18/34), respectively. Olofsson *et al.*[18] assessed the perspective of young men 20 years after surgery and they noted that 8 out of 22 had attenuated ejaculation. All these men underwent Byars’ two-stage technique. Bubanj *et al.*[9] documented spraying or dribbling of ejaculate in a third of patients. Miller *et al.*[19] studied the sexual and urinary function of 19 men who had undergone reconstruction for perineal or perineoscrotal hypospadias. Though 15 of them had normal sexual function, only seven had satisfactory ejaculation. In the proximal hypospadias, the neourethral tube is devoid of smooth muscle and corpus spongiosum. Hence there may be little room for improvement in this group with the operative techniques being used now. With the utilization of TIP, urethral plate mobilization and spongioplasty for proximal hypospadias[20] whether there will be an improvement in ejaculation remains to be seen.

### Fertility

Literature is scant on the fertility of men who had hypospadias. Aho *et al.* found that men who had hypospadias during childhood were less likely to live with a partner and that they had fewer children (0.8 vs. 1.1).[10] The difference was not statistically significant. Bracka evaluated the semen analysis of 169 men who were operated for hypospadias in childhood.[21] Of the 32 who had fathered a child and only one had sperm count of < 20 million/ml. But 40 of the 137 (29%) whose fertility was not proven had sperm counts < 20 million/ml. Two recent publications on sexual function following hypospadias have not assessed fertility.[2,5]

## CONCLUSION

Genital perception is mostly unaffected, especially in those with distal hypospadias operated in childhood. An adverse effect on sexual life has been noted, but the results are discordant. Disturbance in sexual performance seems to be attributed to small penile size. Ejaculatory disturbance has been noted in almost all series. There is no convincing evidence for impaired fertility. The operative procedures based on which these studies were conducted have mostly been replaced by modern techniques which are more anatomical. Furthermore, the operations are being performed at an earlier age. When hypospadias reconstruction is performed in early childhood, it essential to keep in mind the possible long-term sexual and reproductive implications and to choose options that are least likely to impair sexual and reproductive functions. The long-term follow-up of newer operative techniques are awaited. Evaluation of psychosexual, erectile, ejaculatory and reproductive function of specific techniques, and for varying degrees of hypospadias will give a better idea of the outcome of various procedures. Using a validated objective scoring system will help compare the results of various techniques.
